# Wood’s Vade-Mecum

**Published:** 1877-12

**Authors:** 


*Wood's Physician's Vade-Mecum and Visiting List.* Arranged and prepared by H. C. Wood, M. D. Philadelphia: J. B. Lippincott & Co.

It contains the metric system of weights and measures, mode of artificial respiration poisons and their antidotes, list of doses and diagrams of motor points for the application of electricity. The last will be found of value in the use of electricity when applied to stimulate any muscle or set of muscles to action.

The memoranda for daily visits is not indexed for particular weeks but left in blank to be filled out as needed. This is a great improvement over most of the visiting lists. It is well bound and printed upon excellent paper. It will be found more useful than the majority of visiting lists. C.

